# *Trypanosoma cruzi* I and IV Stocks from Brazilian Amazon Are Divergent in Terms of Biological and Medical Properties in Mice

**DOI:** 10.1371/journal.pntd.0002069

**Published:** 2013-02-21

**Authors:** Wuelton Marcelo Monteiro, Ana Paula Margioto Teston, Ana Paula Gruendling, Daniele dos Reis, Mônica Lúcia Gomes, Silvana Marques de Araújo, Maria Terezinha Bahia, Laylah Kelre Costa Magalhães, Jorge Augusto de Oliveira Guerra, Henrique Silveira, Max Jean de Ornelas Toledo, Maria das Graças Vale Barbosa

**Affiliations:** 1 Tropical Medicine Foundation Dr. Heitor Vieira Dourado, Manaus, Amazonas, Brazil; 2 University of the State of Amazonas, Manaus, Amazonas, Brazil; 3 Federal University of Amazonas, Manaus, Amazonas, Brazil; 4 State University of Maringá, Maringá, Paraná, Brazil; 5 Federal University of Ouro Preto, Ouro Preto, Minas Gerais, Brazil; 6 Hygiene and Tropical Medicine Institute, Center of Malaria and other Tropical Diseases, New University of Lisbone, Lisbone, Portugal; 7 Nilton Lins Universitary Center, Manaus, Amazonas, Brazil; Universidad Centroamericana, Nicaragua

## Abstract

**Background:**

In the Brazilian Amazon, clinical and epidemiological frameworks of Chagas disease are very dissimilar in relation to the endemic classical areas of transmission, possibly due to genetic and biological characteristics of the circulating *Trypanosoma cruzi* stocks. Twenty six *T. cruzi* stocks from Western Amazon Region attributed to the TcI and TcIV DTUs were comparatively studied in Swiss mice to test the hypothesis that *T. cruzi* clonal structure has a major impact on its biological and medical properties.

**Methodology/Principal Findings:**

Seventeen parameters were assayed in mice infected with 14 *T. cruzi* strains belonging to DTU TcI and 11 strains typed as TcIV. In comparison with TcI, TcIV stocks promoted a significantly shorter pre-patent period (p<0.001), a longer patent period (p<0.001), higher values of mean daily parasitemia (p = 0.009) and maximum of parasitemia (p = 0.015), earlier days of maximum parasitemia (p<0.001) and mortality (p = 0.018), higher mortality rates in the acute phase (p = 0.047), higher infectivity rates (p = 0.002), higher positivity in the fresh blood examination (p<0.001), higher positivity in the ELISA at the early chronic phase (p = 0.022), and a higher positivity in the ELISA at the late chronic phase (p = 0.003). On the other hand TcI showed higher values of mortality rates in the early chronic phase (p = 0.014), higher frequency of mice with inflammatory process in any organ (p = 0.005), higher frequency of mice with tissue parasitism in any organ (p = 0.027) and a higher susceptibility to benznidazole (p = 0.002) than TcIV. Survival analysis showing the time elapsed from the day of inoculation to the beginning of the patent period was significantly shorter for TcIV strains and the death episodes triggered following the infection with TcI occurred significantly later in relation to TcIV. The notable exceptions come from positivity in the hemocultures and PCR, for which the results were similar.

**Conclusion/Significance:**

*T. cruzi* stocks belonging to TcI and TcIV DTUs from Brazilian Amazon are divergent in terms of biological and medical properties in mice.

## Author Summary

Chagas disease is caused by the protozoan parasite *Trypanosoma cruzi*, constituting an important health problem in the American Continent. In the Brazilian Amazon, Chagas disease has been recognized as an emerging problem. There are few studies exploring the genetic and biological framework of stocks of *T. cruzi* from the Western Brazilian Amazon, where Chagas disease has a profile of lower morbidity and mortality, appearing mainly in the chronic latent form. Here, we carried out the biological characterization in mice of *T. cruzi* isolates belonging to TcI and TcIV DTUs from the State of Amazonas, Western Brazilian Amazon. *T. cruzi* stocks belonging to TcI and TcIV DTUs from Brazilian Amazon are divergent in terms of biological and medical properties in mice, with a higher virulence for the latter DTU as revealed by several biological parameters. Results strongly support the working hypothesis that biological differences are proportional to the evolutionary divergence among the DTUs, and highlight the need to take into account the phylogenetic diversity of *T. cruzi* natural stocks circulating in the emergent areas for Chagas disease in all applied studies dealing with clinical diversity of Chagas disease, immunology, diagnosis, prognosis, and drug and vaccine trials.

## Introduction

Chagas disease is an important health problem, affecting 8–9 million individuals, with approximately 50,000 new cases annually, in Central and Latin America [1]. The illness is caused by *Trypanosoma cruzi* and has a variable clinical course that may include symptomless infection, overwhelming acute disease or a severe chronic condition. The latter may be hallmarked by cardiovascular and/or gastrointestinal involvement. In the search for improved sources of income, agriculture, livestock rearing, and other socioeconomic activities, human populations began migrating into the natural wild habitats where *T. cruzi* infection was enzootic [2]. Currently, poverty, poor housing and sub-standard living conditions and deforestation promote an incipient adaptation of triatomine vectors to both humans and domestic animals, with increased efficiencies of the wild, domestic, and peridomestic cycles of *T. cruzi* transmission in the areas where Chagas disease emerges [2], [3]. Furthermore, oral transmission through contaminated food is now considered an important route of transmission [4].

*Trypanosoma cruzi* is a heterogeneous taxon with multiple hosts, vectors and routes of infection (for a review, see [5]). Multilocus genotyping consistently reveals six ‘discrete typing units’ (DTUs) [6], named as TcI to TcVI in the last *T. cruzi* nomenclature consensus held in Brazil [7]. TcII, TcV and TcVI predominate in the domestic transmission cycles in the South Cone of South America, where patients may present severe acute disease with chronic cardiac and/or digestive involvement [8]. In the Brazilian Amazon, Venezuela, Colombia, Central and North America, TcI is the predominant DTU and the major cause of both acute and cardiac Chagas' disease, but also is reported from chagasic patients sporadically throughout the Southern Cone [9]–[12]. Unexpectedly, TcI was reported beside TcII, TcIII, TcIV and TcV strains in Mexico, at a relatively high frequency in *Triatoma dimidiata* [13]. TcIII is infrequent from domestic sources and circulates in terrestrial transmission cycles associated with *Dasypus novemcinctus* mainly throughout South America, causing at least three reported cases of Chagas' disease [14]–[16]. TcIV is found in Amazonia and Southern North America, most frequently isolated from primates and recently this DTU was associated to Chagas' disease in the Brazilian Amazon [17] and Venezuela [18].

To understand the diverse phenotypic differences among different *T. cruzi* strains and the potential connection between that variability and different manifestations of Chagas disease, it is essential to have a correct reconstruction of the evolutionary history of *T. cruzi*. There is solid evidence that *T. cruzi* genetic structure is the outcome of recombination events detected as the presence of naturally occurring hybrids [19], mitochondrial introgression [17], [19] and a capacity for genetic exchange in the laboratory [20], occurring more commonly within epidemiologically linked strains [21]. Likewise, clonal diversity among humans, reservoir hosts and vectors suggested complex patterns of superinfection and/or coinfection in oral and vector-borne Chagas' disease cases [22]. Advances in the comprehension of the molecular epidemiology of Chagas' disease and geographic distribution of the six DTUs were not accompanied by a similar effort to straightforwardly elucidate the biological framework of the stocks and clones studied both in the vertebrate hosts and vectors. Some ancient studies have confirmed that *T*. *cruzi* genetic diversity is correlated with the intrinsic characteristics of the parasite such as the behavior in vitro [23], [24], in the vector [25], and in the vertebrate host, namely virulence [26], pathological alterations and tropism toward specific organs [26], [27].

In the Brazilian Amazon Region, Chagas' disease has been recognized as an important and emerging problem. The epidemiological situation of Chagas' disease in this region, where enzootic *T. cruzi* transmission cycles involve a great diversity of vectors and reservoir hosts [4], [28], suitably illustrates the concerns about the consolidation of Chagas' disease control. Adventitious adult triatomines maintain continuous, low-intensity transmission in rural settings; as a result, human infection is hypo-endemic in the region, with about 100,000 to 300,000 people chronically carrying *T. cruzi* [4]. Sylvatic triatomines are also involved in localized disease outbreaks related to oral *T. cruzi* transmission via contaminated foodstuffs, and account for the relatively high infection prevalence (4–5%) reported among extractivist forest workers such as piaçava palm fiber collectors [4], [28]. In this region a few cases of chronic Chagas' disease were reported, appearing uniquely as miocardiopathy without records of digestive tract pathology [29]. So far there is no information about the DTUs responsible for chronic cases in the Brazilian Amazon. Isolates from patients with acute Chagas' disease were typed as TcI and Z3 (TcIII or TcIV) in the state of Pará [30] and as TcIV in two outbreaks in the state of Amazonas [17].

The diverse clinical outcome of human *T. cruzi* infection has been attributed to the genetic heterogeneity of parasite populations and to the host's genetic background [31]. Miles et al. [9] have suggested that the variability of clinical manifestations of the disease could be in part explained by the parasite's genetic diversity. Knowledge on the parallel evolution between biological differences and genetic divergence among *T. cruzi* natural stocks is highly desirable because it may play an important role in comparative researches on the clinico-epidemiological and pathogenic perspectives. Predicting the clinical outcome of an infection based on the infecting DTU (and to what extent it is possible to do) is an important and worthy goal that needs previous comparative biology studies. The aim of this work is to carry out the biological characterization of *T. cruzi* isolates belonging to TcI and TcIV DTUs from the State of Amazonas, Western Brazilian Amazon.

## Methods

### Ethics statement

The use of stocks of *T. cruzi* obtained from patients has been approved by the Ethics in Research on Humans Committee of the Tropical Medicine Foundation of the State of Amazonas (approval number 360/07). Patients diagnosed with Chagas disease were treated according to the guidelines of the Brazilian Health Ministry. We obtained written informed consent from all participants involved in our study. Marsupials' captures and handling for blood sample collection were performed according to permits from the Brazilian Institute for Environment (approval number 1830651/07). Use of mice in this study and all the procedures using these animals were approved by the Ethics in Research on Animal Committee of the State University of Maringá (approval number 113/09). The procedures with the animals followed the ethical principles for animal use in research according to the Brazilian College of Animal Experimentation.

### Parasites stocks

**Figure 1:**
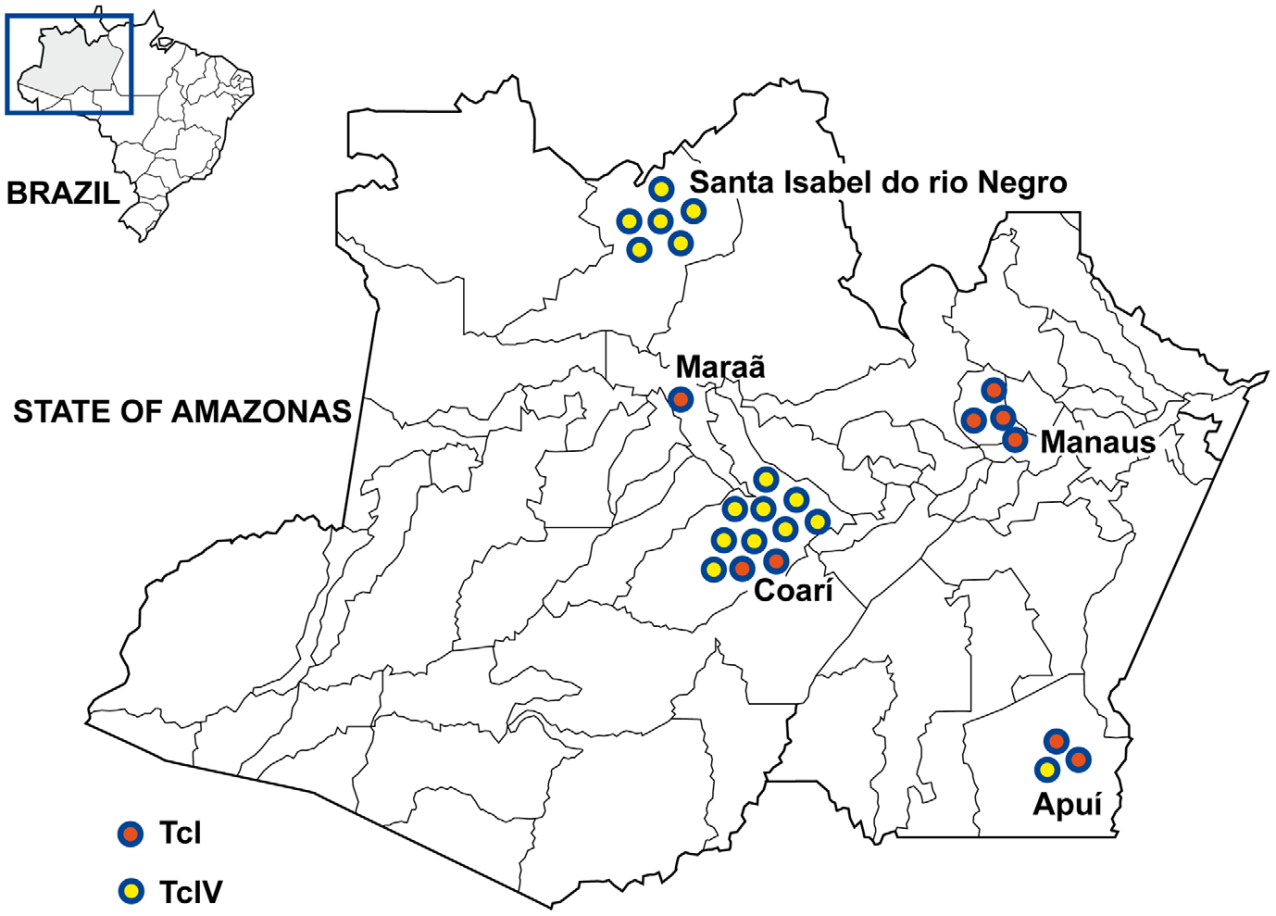
Geographic origin of the *Trypanosoma cruzi* strains from the State of Amazonas, Brazil.

All the stocks biologically characterized in this investigation were isolated from municipalities distantly located in the State of Amazonas (Fig. 1) and were genotyped previously as TcI or TcIV [17]. Molecular characterization was performed by PCR of the mini-exon [32] and ribosomal RNA [33] genes and by sequencing of the mitochondrial cytochrome c oxidase subunit II [34] and glucose-phosphate isomerase [20] genes. According to the *T. cruzi* nomenclature consensus [7], we included a set of fourteen stocks belonging to DTU TcI and a set of eleven stocks of the DTU TcIV. Information on the laboratory code, host, method of isolation, geographic origin, and DTU of these stocks is given in Table S1. For more details about these procedures see Monteiro et al. [17].

The methods used in this work were summarized in the Figure S1.

### Inoculation of mice

For each *T. cruzi* stock, a group of 20 male Swiss mice aged 21–28 days and weighting between 18 g and 20 g was used. All the animals were supplied by the central bioterium of the State University of Maringá and were kept under suitable temperature, humidity, and availability of water and food *ad libitum*. Inoculation was performed intraperitoneally and the inoculum was calculated according to Brener [35], ranging from 2,8×10^3^ to 1.0×10^4^ blood trypomastigotes per animal (Table S1). For experiments with thirteen stocks presenting subpatent parasitemia in Swiss mice, inoculum of metacyclic trypomastigotes from late-stationary-phase culture in LIT medium [36] was employed. We used an inoculum of 1,0×10^6^ metacyclic trypomastigotes per animal for histopathological studies. The number of parasites was calculated by using a Neubauer chamber, with the subsequent calculation of the percentage of trypomastigotes in random counts of 500 forms on a Giemsa-stained slide (Table S1).

### Parasitological parameters

The curve of parasitemia was based on the search on trypomastigotes forms in the bloodstream. Fresh blood examination (FBE) was daily carried out from the 3^th^ day after inoculation (d.a.i.) until the test became negative for at least three consecutive days in the case of patent parasitemia or for a 30-day period in the case of subpatent parasitemia. Counting of parasites was performed on 5 µL of blood collected from the animal's tail as described by Brener [35]. In addition to determining the percentage of animals presenting positive FBE (%+FBE), mean pre-patent period (PPP), mean patent period (PP), mean daily parasitemia (MDP), maximum peak of parasitemia (Pmax) and day of maximum peak of parasitemia (DPmax) were obtained for each DTU [26].

### Hemoculture (HC)

On the 55^th^ d.a.i., 0.5 mL of blood was aseptically collected from the retro-orbital plexus and placed in two tubes containing 3 mL of LIT medium each, according to Filardi and Brener [37]. The cultures were kept at 28°C and monitored with microscopy for parasite growth at 30, 45, and 60 days after seeding. This technique allowed the percentage of mice presenting positive hemoculture (%+HC) to be obtained for each DTU.

### Polymerase Chain Reaction (PCR)

For PCR, blood samples were obtained at the same time that HC was carried out. Two hundred microliters of blood from each animal was added to double the volume of 6.0 M/0.2 M guanidine/EDTA and stored at room temperature. The lysate was boiled for 7 min and the DNA extraction carried out in an aliquot of 100 µL using the Wincker et al. [38] protocol as modified by Gomes et al. [39]. The DNA solution was then washed with 70% ethanol prior to precipitation in order to remove potential PCR inhibitors. Finally, the DNA was re-suspended in 10 µL H_2_O MiliQ. During this stage, one negative and one positive control were added to each group of four samples. PCR amplification was performed in a total volume of 11 µL, using 121 and 122 primers to amplify a specific fragment of 330 base pairs (bp) of kinetoplast DNA (kDNA) of *T. cruzi*. The reaction mixture was submitted to 35 amplification cycles with a thermocycler (Techne TC-512, UK), using 95°C for 1 min for denaturation with a longer initial time of 5 min, 65°C for 1 min for primer annealing, and 72°C for 1 min for extension with a final incubation at 72°C for 10 min. In this step, as a contamination control, two negative and two positive controls from the extraction step were added to every eight samples, and one negative and one positive control to the PCR. All the stages were carried out in separate environments with reagents, materials, and equipments exclusive to each area. The PCR products were visualized in 4.5% polyacrylamide silver-stained gel.

With the PCR results was obtained the parameter percentage of mice with positive PCR (%+PCR) for each DTU [40].

### Infectivity and mortality

The infectivity rate (%INF) was calculated as the percentage of animals presenting positive FBE within the first two months following inoculation and/or positive HC and/or PCR on the 55^th^ d.a.i. Cumulative mortality (%MOR) was recorded every day during the infection course, being calculated as the percentage of deaths observed in the acute (from inoculation until the 90^th^ d.a.i) and chronic phases (from the 90^th^ until the 180^th^ d.a.i.). Day of maximum mortality (DMM) was determined by the mean of the day when occurred the deaths for each DTU.

### Enzyme-linked immunosorbent assay (ELISA)

An ELISA modified according to Voller et al. [41] was used. Alkaline antigen of the *T. cruzi* Y (TcII) strain obtained in the exponential growth phase in LIT medium, and peroxidase-labeled anti-mouse immunoglobulin G conjugated (Bethyl Laboratories, Montgomery, AL, USA) were used. Samples of serum collected in the early chronic phase (115^th^ d.a.i.) and in the late chronic phase (205^th^ d.a.i.) and diluted to 1∶100 in phosphate-buffered saline, were used. The mean absorbance for 10 negative-control serum samples plus 2 standard deviations was used as the cut-off to discriminate positive and negative results.

With the ELISA results was obtained the parameter percentage of mice with positive ELISA (%+ELISA) for each DTU [40].

### Susceptibility to benznidazole

After inoculation the animals were divided into two groups: 10 treated (T) and 10 untreated controls (NT). The first group was treated orally with daily doses of benznidazole (Lafepe, Brazil) 100 mg/kg of body weight for 20 consecutive days, starting of the 5th day after inoculation [37]. From the 3^rd^ d.a.i. mice were subjected daily to FBE to confirm the infection and parasitemia record before treatment starting. Benznidazole was suspended in distilled water using Arabic gum and this suspension was administered by gavage.

Both T and NT groups were submitted to different diagnostic techniques for cure monitoring and determination of susceptibility to BZ. Mice with negative FBE, HC, PCR and ELISA results after completed treatment were considered cured [42]. Those with at least one positive result in any test were considered non-cured. The procedures for conducting these tests are the same as described in previous items.

The cure rate in animals inoculated with each stock and treated with benznidazole was obtained by the ratio between the number of animals considered cured and the total treated animals ×100. To determine the in vivo susceptibility of *T. cruzi* stocks to the chemotherapeutic agent was adopted criterion similar to Toledo et al. [42]: stocks with cure rates of 0% to 33% were resistant to the drug, those with cure rates between 34% and 67% were considered intermediate sensitivity and stocks with cure rates above 67% were considered sensitive to the drug.

### Histopathological parameters

Histopathological analysis was carried out on animals from seven experiments with strains typed as TcI (AM28, AM38, AM41, AM49 and AM61) and TcIV (AM69 and AM70) using seven mice for each experiment. Infection was confirmed by FBE and/or HC. For each *T. cruzi* stock, all infected surviving mice were necropsied on the 90^th^ d.a.i. for histopathological studies. The following organs and tissues were collected: (1) heart, (2) skeletal muscle, (3) lungs, (4) large intestine, (5) brain, (6) liver and (7) spleen. This material was routinely processed and embedded in paraffin. Five-micrometer sections of three blocks containing every organ of each mouse were obtained for further evaluation. These preparations were stained with hematoxylin-eosin for microscopic examination. The tissue parasitism (TP) and inflammatory process (IP) displayed by different organs or tissues were classified as absent, mild, moderate or severe. With these results was obtained the frequency of mice with inflammatory process and tissue parasitism for each DTU.

### Statistical analysis

Data were analyzed using SPSS version 16.0 for Windows (SPSS Inc., Chicago, IL, USA). Normal distribution of data was evaluated with the Kolmogorov-Smirnov test. Chi-square or Fisher's test was used to test differences in proportions, and Student t test was used to test differences in means. Levene's test was used to assess the equality of variances in different samples (DTUs). A Kaplan-Meier survival analysis was performed in order to detect differences in the time elapsed from the day of inoculation to the death episodes and the beginning of the patent period between mice inoculated with TcI and TcIV DTUs of *T. cruzi*. Log-rank test was used to test differences. Statistical significance was considered if p<0.05.

## Results

**Figure 2:**
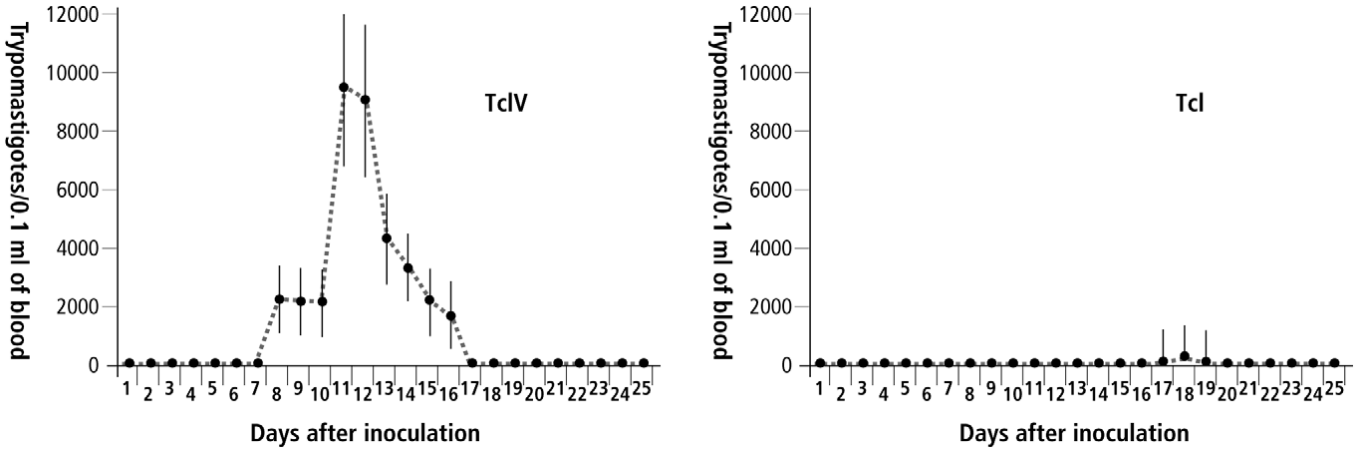
Curves of mean parasitemia obtained from *Trypanosoma cruzi* I and IV strains.

Significant mean and frequency differences are apparent for biological properties in Swiss mice between the TcI and TcIV stocks. The statistical comparison evidenced that 15 out 17 parameters demonstrated significant differences between the DTUs (Tables 1 and 2). TcIV stocks promoted a significantly shorter PPP (p<0.001), a longer PP (p<0.001), higher values of MDP (p = 0.009) and Pmax (p = 0.015), and an earlier DMP (p<0.001) and DMM (p = 0.018), in comparison with TcI. A great standard deviation was observed in these biological parameters, mainly in PP, MDP, Pmax, and DMM showing that stocks of the same genetic lineage were not homogeneous in their characteristics (Table 1). Figure 2 shows how dissimilar are the curves of mean parasitemia built for TcI and TcIV strains and highlights the higher and earlier parasitemia observed for TcIV. Area under the curve was significantly greater for the set of TcIV strains (p<0.001).

**Table 1:** Mean values of biological parameters from *Trypanosoma cruzi* I and IV strains.

	Discrete typing unit (DTU)		
Parameter	TcI	TcIV	p(b)
Mean pre-patent period (in days)	12.9±0.7	5.3±2.4	**<0.001**
Mean patent period (in days)	0.2±0.6	4.6±2.9	**<0.001**
Mean daily parasitemia(a)	10.3±33.3	1,484.3±2,543.8	**0.009**
Peak of maximum parasitemia(a)	143.7±356.6	10,971.7±3,112.2	**<0.001**
Day of maximum parasitemia	15±0.8	7.4±0.5	**<0.001**
Day of maximum mortality	134±47.3	28.9±29.2	**0.018**
Number of significant differences			6/6

a Number of trypomastigotes/0,1 mL of blood.

b p<0.05: significant difference in the T-student test.

**Table 2:** Virulence parameters obtained in Swiss mice from *Trypanosoma cruzi* I and IV strains.

	Discrete typing unit (DTU)		
Parameter	TcI	TcIV	p(f)
Mortality rate in the acute phase (%)(a)	2.4	12.3	**0.047**
Mortality rate in the chronic phase (%)(b)	9.8	1.2	**0.014**
Infectivity rate (%)(c)	64.9	84.4	**0.002**
Mice with positive fresh blood examination (%)	7.9	79.5	**<0.001**
Mice with positive hemoculture (%)	64.7	49.4	0.061
Mice with positive PCR (%)	60.9	45.6	0.109
Mice with inflammatory process in any organ (%)	81.3	28.6	**0.005**
Mice with parasitism in any organ (%)	12.5	0.0	**0.027**
Susceptibility to benznidazole (%)	80.6	57.0	**0.002**
%+ELISA in the early chronic phase(d)	53.8	82.8	**0.022**
%+ELISA in the late chronic phase(e)	20.0	87.5	**0.003**
Number of significant differences	9/11		

a Cumulative mortality from inoculation until the 90^th^ day after inoculation (d.a.i);

b Cumulative mortality from the 90^th^ until the 180^th^ d.a.i.;

c Percentage of animals presenting positive fresh blood examination within the first two months following inoculation and/or positive hemoculture and/or PCR on the 55^th^ d.a.i.;

d Percentage of animals presenting positive ELISA in the 115^th^ d.a.i.;

e Percentage of animals presenting positive ELISA in the 205^th^ d.a.i..

f p<0.05: significant difference; N.S.: not significant.

In the Table 2 are seen that TcIV stocks showed higher values of %MOR in the acute phase (p = 0.047), %INF (p = 0.002), %+FBE (p<0.001), %+ELISA in the early chronic phase (p = 0.022), and %+ELISA in the late chronic phase (p = 0.003) than the TcI strains. On the other hand TcI showed higher values of %MOR in the chronic phase (p = 0.014), frequency of mice with inflammatory process in any organ (p = 0.005), frequency of mice with tissue parasitism in any organ (p = 0.027) and susceptibility to benznidazole (p = 0.002) than TcIV. The notable exceptions come from parameters obtained through amplification methods of diagnosis (%+HC and %+PCR), for which no result is statistically significant (p<0.05) (Table 2). Stocks presenting resistance to benznidazole were found uniquely within TcIV stocks (3/10). Tissue parasitism and severe or moderate inflammatory process in the early chronic phase were recorded only in mice inoculated with TcI strains (Table 3). Amastigote nests were observed only in heart tissue and inflammatory process was observed in heart, skeletal muscle and brain.

**Table 3:** Number of mice infected with *Trypanosoma cruzi* I and IV strains presenting histopathological alterations.

Parameter	Intensity	DTU	
		TcI (n = 32)	TcIV (n = 7)
Tissue parasitism	Mild	4	0
	Absent	28	7
Inflammatory process	Severe	7	0
	Moderate	12	0
	Mild	7	2
	Absent	6	5

**Figure 3:**
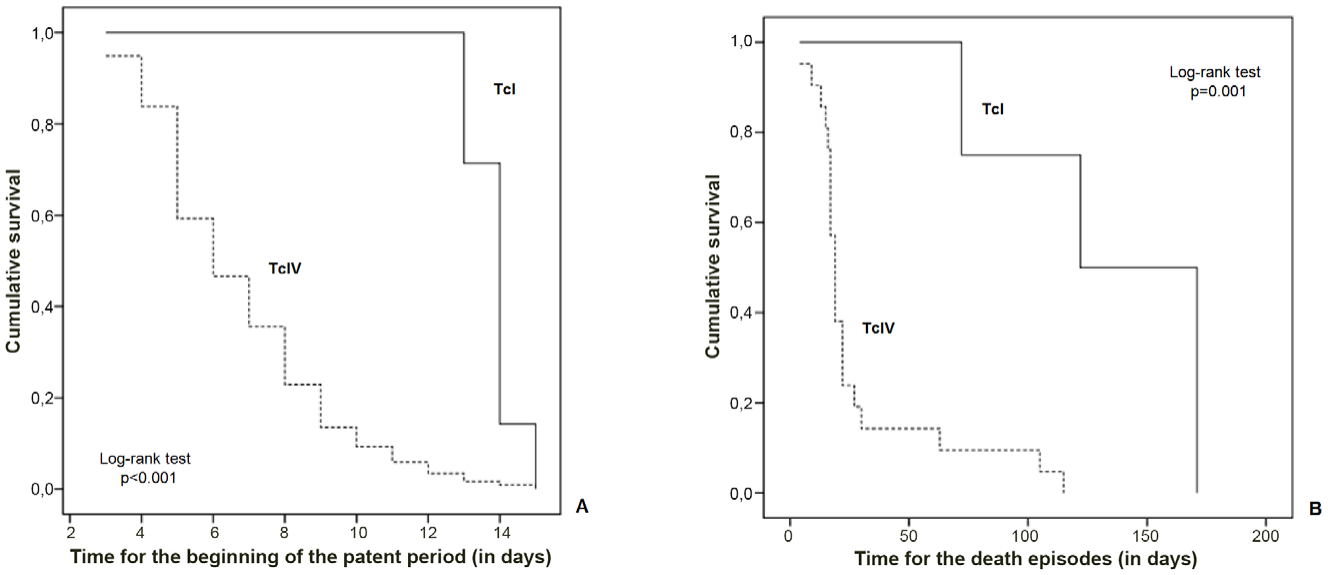
Survival analysis using patent period and time until death episodes for TcI and TcIV strains. Panel A shows a Kaplan-Meier survival analysis performed in order to detect differences in the time elapsed from the day of inoculation to the death episodes and Panel B shows the same analysis from the day of inoculation to the beginning of the patent period, in mice inoculated with TcI and TcIV DTUs of *T. cruzi*. Log-rank test was used to test differences.

Kaplan-Meier survival analysis showing the time elapsed from the day of inoculation to the beginning of the patent period was significantly shorter for TcIV strains and the death episodes triggered following the infection with TcI occurred significantly later in relation to TcIV (Figure 3).

## Discussion

Several studies of the properties of *T. cruzi* in cell and acellular cultures [23], [24], [43], virulence and pathogenicity in mice [23], [26], [44], [45], development in vectors [25], [46] and response to specific chemotherapy [24], [42], [47], [48] support a strong association between genetic distance and the biological and medical properties of the parasite. The above-mentioned studies have brought significant contributions, but none of them relied on a population genetic framework representative of the emergent Chagas disease areas, represented in Brazil by the Amazon region. Furthermore, to our knowledge, this is the first investigation focusing on biological and medical properties of TcIV strains from Amazon Region. Human acute Chagas disease caused by this DTU has been described [15] and recently, our group reported that the majority of the acute cases in the Western Brazilian Amazon, including outbreaks by oral transmission, were triggered by TcIV [17].

In this work TcIV showed clearly higher virulence in comparison with TcI strains, as demonstrated by the higher values of parasitemia parameters and shortest PPP. TcI strains promoted predominantly subpatent and intermittent parasitemias, in agree with the results obtained from previous experimental studies using TcI from the Amazon region [49]. Furthermore, TcIV parasites were more infective for mice and promoted higher mortality in the acute phase, while TcI led to higher mortality in the chronic phase. However, taking into account the mortality parameter, we suggest that both DTUs had low virulence, since a previous report demonstrated that *T. cruzi* stocks (both TcI and TcII) isolated from patients in the acute phase killed 100% of the animals [50].

Previous reports have shown a great competence of TcII strains to invade human and monkey cells [51], [52], to infect and render patent parasitemias in mice [53] and to infect and multiplicate inside macrophages [54] in comparison to TcI strains. However, it has to be mentioned that some discrepant results were reported as well. Revollo et al. [24], for instance, showed that TcI parasites displayed a higher infection rate to Vero cell monolayers than TcII stocks. Some works demonstrated that TcI strains have a greater ability to complete its life cycle in the vector [25], [46], and a higher capacity for intracellular multiplication in the vertebrate host populations, leading to higher parasitemias in comparison to TcII [26], [45], [55], [56]. In a study conducted with TcI and TcIV isolates from United States, the two *T*. *cruzi* DTUs caused differential infection dynamics in mice and rats as determined by PCR detection of *T*. *cruzi* DNA in the blood and tissues [57]. These authors found that sylvatic TcI isolates from the United States had greater infectivity to laboratory rodents than TcIV isolates, contradicting our results with isolates from Brazilian Amazon. Despite being indistinguishable by traditional genotyping and generally being assigned to Z3, there are evidences that TcIV from South America and TcIV from North America correspond to independent lineages using cytochrome b and SSU rDNA sequences, which circulate in distinct hosts and ecological niches [58].

The histopathological results revealed in general the presence of few amastigotes nests and mild inflammatory lesions. TcI triggered inflammatory process in skeletal muscle, heart and brain, while TcIV promoted mild inflammatory process uniquely in skeletal muscle. Parasitism was observed only in 4 mice examined, all infected by two TcI strains (AM49 and AM61), that presented well-limited pseudocysts surrounded by mild inflammatory reaction. Moreover, severe inflammatory process occurred only in skeletal muscle of mice infected by TcI. Histopathological alterations were not observed in large intestine, liver and, spleen of infected mice. Thus, although the lower values of parasitemia, TcI determined relatively most severe inflammatory process and higher frequency of tissue parasitism in mice than TcIV.

Although TcIV stocks have been demonstrated higher virulence than TcI, its pathogenicity was also incipient. These data taken together emphasize that the biological and medical properties of the stocks from this area are crucial to be considered in researches about the clinical end epidemiological picture of Chagas disease in the Amazon context. These profiles agree with the hypothesis of authors who suggested that the framework of chronic latent infection and a relative low performance of diagnostic tests in the State of Amazonas are due to the lower parasitemia, virulence, and pathogenicity of the wild parasites in this area [29]. However, pathogenicity triggered by TcIV should be interpreted with caution, since there were documented cases of chronic Chagas heart disease caused by TcI/TcIV mixed infections in Colombia [59], and two cases of chronic disease with cardiac/digestive involvement associated with Z3 parasite that clustered with CANIII (prototype of the TcIV DTU) at the isoenzymic analysis in Ecuador [60]. In the Western Brazilian Amazon, TcIV has been associated with two outbreaks of acute Chagas disease probably linked to oral transmission [17].

Resistance to benznidazole was significantly higher for TcIV strains. In vitro [24], [61] and in vivo [42], [47], [48] studies found that clones belonging to the lineage TcI are more resistant to benznidazole and nifurtimox than that belonging to the lineage TcII. Actually, as observed in central Brazil, patients infected with TcI strains showed higher resistance to chemotherapy with benznidazole compared with those infected with TcII [62]. Murta et al. [63] reported higher efficiency of chemotherapy with benznidazole and nifurtimox in human disease in areas with high prevalence of hybrid zymodemes. However, preliminary data about the response to specific chemotherapy in patients from outbreaks related to oral transmission in areas of predominance of TcI in the Amazon Region have been shown high parasitological cure rates and decrease of the antibody titres [30], [64], reinforcing that TcI isolates from Brazilian Amazon poses a dissimilar behavior when compared to other areas.

The seminal biological studies have demonstrated the huge heterogeneity of *T. cruzi* parasites, but were probably hampered by the fact that recent and unexpanded isolates from wild environment were not frequently used. Our work includes non- or low-passaged parasite natural stocks and contributes to diminish this biased sampling. A limitation of this approach may be the instability of the composition of these stocks, but we believe that our methodology better simulates what happens in nature, where populations are often identified in multiclonality in vertebrate hosts and triatomines [5]. Results strongly support the working hypothesis that biological differences are proportional to the evolutionary divergence among the DTUs. This shows that evolutionary divergence and biological differences do not evolve independently, and can be statistically predicted from each other to a large extent. We highlight the need to take into account the phylogenetic diversity of *T. cruzi* natural stocks circulating in the emergent areas for Chagas disease in all applied studies dealing with clinical diversity of Chagas disease, immunology, diagnosis, prognosis, and drug and vaccine trials. The potential of the two lineages in terms of severity and clinical manifestations remains to be determined in humans, namely for TcIV strains.

## Supporting Information

Figure S1 Methods of the study.
(PPTX)

Table S1 Characteristics of *Trypanosoma cruzi* stocks from the State of Amazonas, Brazil.
(DOC)
