# Supplementary material for: Trypanosoma cruzi I and IV Stocks from Brazilian Amazon Are Divergent in Terms of Biological and Medical Properties in Mice
Source: PLoS Negl Trop Dis. 2013 Feb 21;7(2):e2069. doi: 10.1371/journal.pntd.0002069 (PMC3578774; doi:10.1371/journal.pntd.0002069)
Supplement: Figure S1 — Methods of the study. (PPTX) [file pntd.0002069.s001.pptx]

## Slide 1
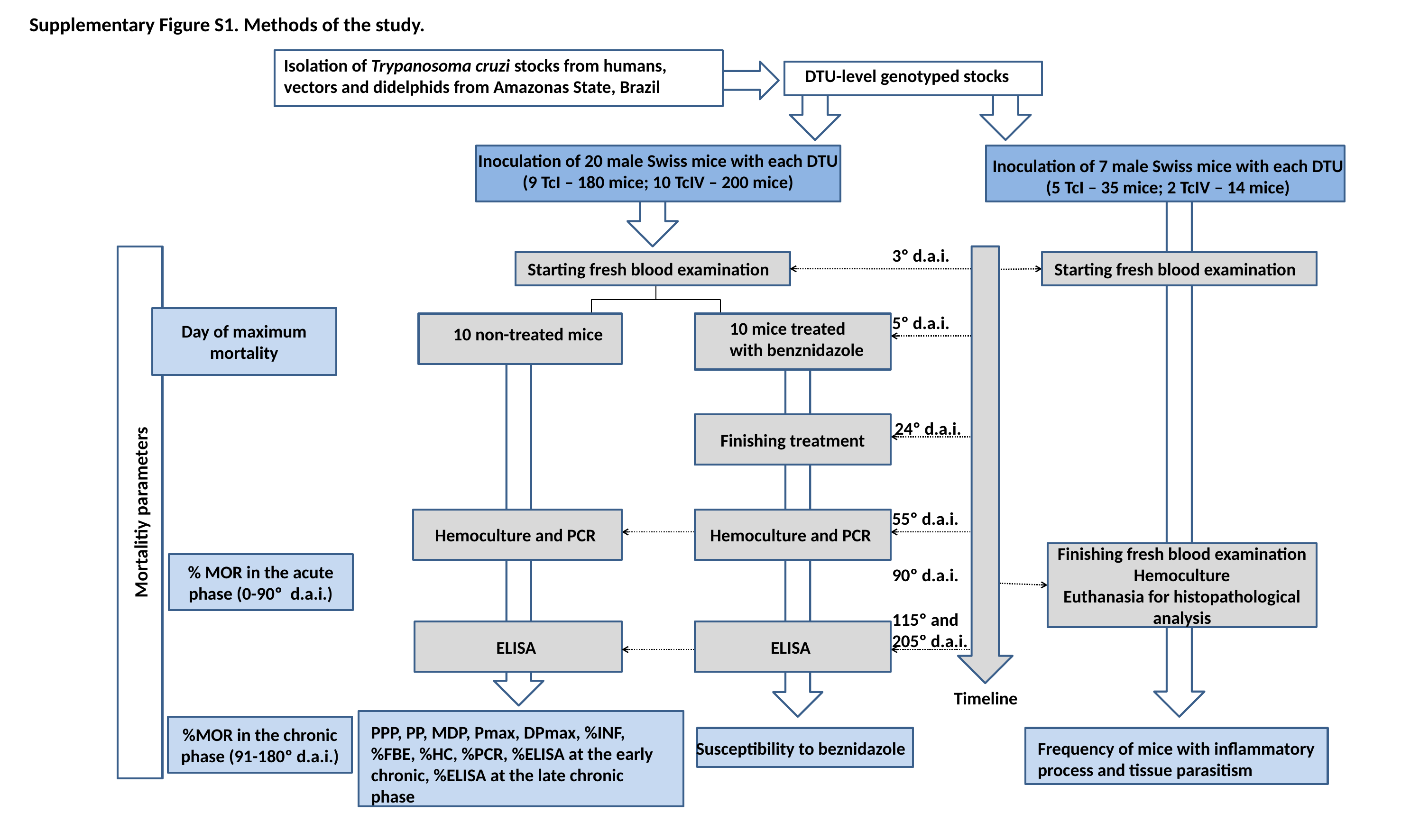

Supplementary Figure S1. Methods of the study.
Isolation of Trypanosoma cruzi stocks from humans, vectors and didelphids from Amazonas State, Brazil
DTU-level genotyped stocks
Inoculation of 20 male Swiss mice with each DTU
 (9 TcI – 180 mice; 10 TcIV – 200 mice)
Inoculation of 7 male Swiss mice with each DTU
 (5 TcI – 35 mice; 2 TcIV – 14 mice)
3º d.a.i.
Starting fresh blood examination
Starting fresh blood examination
Day of maximum mortality
5º d.a.i.
10 mice treated
with benznidazole
10 non-treated mice
24º d.a.i.
Finishing treatment
Mortalitiy parameters
55º d.a.i.
Hemoculture and PCR
Hemoculture and PCR
Finishing fresh blood examination Hemoculture
Euthanasia for histopathological analysis
% MOR in the acute phase (0-90º d.a.i.)
90º d.a.i.
115º and
205º d.a.i.
ELISA
ELISA
Timeline
%MOR in the chronic phase (91-180º d.a.i.)
PPP, PP, MDP, Pmax, DPmax, %INF, %FBE, %HC, %PCR, %ELISA at the early chronic, %ELISA at the late chronic phase
Susceptibility to beznidazole
Frequency of mice with inflammatory process and tissue parasitism
