# Supplementary material for: Trypanosoma cruzi I and IV Stocks from Brazilian Amazon Are Divergent in Terms of Biological and Medical Properties in Mice
Source: PLoS Negl Trop Dis. 2013 Feb 21;7(2):e2069. doi: 10.1371/journal.pntd.0002069 (PMC3578774; doi:10.1371/journal.pntd.0002069)
Supplement: Table S1 — Characteristics of Trypanosoma cruzi stocks from the State of Amazonas, Brazil. (DOC) [file pntd.0002069.s002.doc]

Supporting information (Table S1). Characteristics of *Trypanosoma cruzi* stocks from the State of Amazonas, Brazil.

| **International nomenclature *^(a)^*** | **Laboratory code** | **Municipality of origin** | **Method of isolation** | **Host** | **Inoculum** | **DTU** |
| --- | --- | --- | --- | --- | --- | --- |
| MDID/BR/2007/AM28 | AM28 | Manaus | Hemoculture | *Didelphis marsupialis* | 1,0 x 10^6 (^*^d^*^)*^ | TcI |
| MDID/BR/2007/AM30 | AM30 | Manaus | Xenodiagnosis | *Didelphis marsupialis* | 1,0 x 10^4 (^*^c^*) | TcI |
| MDID/BR/2007/AM31 | AM31 | Manaus | Xenodiagnosis | *Didelphis marsupialis* | 2,0 x 10^6 (^*^d^*) | TcI |
| MDID/BR/2007/AM32 | AM32 | Manaus | Hemoculture | *Didelphis marsupialis* | 2,8 x 10^3 (^*^c^*) | TcI |
| TPIS/BR/2007/AM33 | AM33 | Manaus | Xenoculture | *Rhodnius pictipes* | 2,0 x 10^6 (^*^d^*) | TcI |
| MDID/BR/2007/AM34 | AM34 | Manaus | Hemoculture | *Didelphis marsupialis* | 2,0 x 10^6 (^*^d^*) | TcI |
| TROB/BR/2007/AM37 | AM37 | Coari | Xenoculture | *Rhodnius robustus* | 2,0 x 10^6 (^*^d^*) | TcI |
| MPHI/BR/2007/AM38 | AM38 | Coari | Hemoculture | *Philander opossum* | 1,0 x 10^6 (^*^d^*^)*^ | TcI |
| MDID/BR/2007/AM39 | AM39 | Manaus | Hemoculture | *Didelphis marsupialis* | 2,8 x 10^3 (^*^c^*) | TcI |
| TROB/BR/2007/AM41 | AM41 | Maraã | Xenoculture | *Rhodnius robustus* | 1,0 x 10^6 (^*^d^*^)*^ | TcI |
| MDID/BR/2008/AM44 | AM44 | Manaus | Hemoculture | *Didelphis marsupialis* | 2,0 x 10^6 (^*^d^*) | TcI |
| MHOM/BR/2008/AM49 | AM49 | Coari | CSF (*^b^*) culture | Human – acute phase | 1,0 x 10^6 (^*^d^*^)*^ | TcI |
| TROB/BR/2009/AM56 | AM56 | Apuí | Inoculation in mice | *Rhodnius robustus* | 2,0 x 10^6 (^*^d^*) | TcI |
| TPIS/BR/2009/AM61 | AM61 | Apuí | Inoculation in mice | *Rhodnius pictipes* | 1,0 x 10^6 (^*^d^*^)*^ | TcI |
| MHOM/BR/2007/AM05 | AM05 | Coari | Hemoculture | Human – acute phase | 1,0 x 10^4 (^*^c^*) | TcIV |
| MHOM/BR/2007/AM14 | AM14 | Coari | Hemoculture | Human – acute phase | 1,0 x 10^4 (^*^c^*) | TcIV |
| MHOM/BR/2007/AM15 | AM15 | Coari | Hemoculture | Human – acute phase | 1,0 x 10^4 (^*^c^*) | TcIV |
| MHOM/BR/2007/AM18 | AM18 | Coari | Hemoculture | Human – acute phase | 1,0 x 10^4 (^*^c^*) | TcIV |
| TROB/BR/2009/AM57 | AM57 | Apuí | Inoculation in mice | *Rhodnius robustus* | 1,0 x 10^4 (^*^c^*) | TcIV |
| MHOM/BR/2009/AM62 | AM62 | Santa Isabel do Rio Negro | Hemoculture | Human – acute phase | 1,0 x 10^4 (^*^c^*) | TcIV |
| MHOM/BR/2009/AM64 | AM64 | Santa Isabel do Rio Negro | Hemoculture | Human – acute phase | 5,0 x 10^3 (^*^c^*) | TcIV |
| MHOM/BR/2009/AM67 | AM67 | Santa Isabel do Rio Negro | Hemoculture | Human – acute phase | 1,0 x 10^4 (^*^c^*) | TcIV |
| MHOM/BR/2009/AM68 | AM68 | Santa Isabel do Rio Negro | Hemoculture | Human – acute phase | 1,0 x 10^4 (^*^c^*) | TcIV |
| MHOM/BR/2009/AM69 | AM69 | Santa Isabel do Rio Negro | Hemoculture | Human – acute phase | 1,0 x 10^4 (^*^c^*) | TcIV |
| MHOM/BR/2009/AM69 | AM69 | Santa Isabel do Rio Negro | Hemoculture | Human – acute phase | 1,0 x 10^6 (^*^d^*^)*^ | TcIV |
| MHOM/BR/2009/AM70 | AM70 | Coari | Hemoculture | Human – acute phase | 1,0 x 10^6 (^*^d^*^)*^ | TcIV |

*^a^* Based on Anonymous (1999); *^b^* Cerebrospinal fluid; *^c^* Number of blood trypomastigotes/animal; *^d^* Number of metacyclic trypomastigotes in LIT/ animal.

*Strains used only for histopathological studies.
